# Whole Genome Re-Sequencing and Characterization of Powdery Mildew Disease-Associated Allelic Variation in Melon

**DOI:** 10.1371/journal.pone.0157524

**Published:** 2016-06-16

**Authors:** Sathishkumar Natarajan, Hoy-Taek Kim, Senthil Kumar Thamilarasan, Karpagam Veerappan, Jong-In Park, Ill-Sup Nou

**Affiliations:** Department of Horticulture, Sunchon National University, Suncheon, Jeonnam 540–950, Republic of Korea; US Department of Agriculture, UNITED STATES

## Abstract

Powdery mildew is one of the most common fungal diseases in the world. This disease frequently affects melon (*Cucumis melo L*.) and other Cucurbitaceous family crops in both open field and greenhouse cultivation. One of the goals of genomics is to identify the polymorphic loci responsible for variation in phenotypic traits. In this study, powdery mildew disease assessment scores were calculated for four melon accessions, ‘SCNU1154’, ‘Edisto47’, ‘MR-1’, and ‘PMR5’. To investigate the genetic variation of these accessions, whole genome re-sequencing using the Illumina HiSeq 2000 platform was performed. A total of 754,759,704 quality-filtered reads were generated, with an average of 82.64% coverage relative to the reference genome. Comparisons of the sequences for the melon accessions revealed around 7.4 million single nucleotide polymorphisms (SNPs), 1.9 million InDels, and 182,398 putative structural variations (SVs). Functional enrichment analysis of detected variations classified them into biological process, cellular component and molecular function categories. Further, a disease-associated QTL map was constructed for 390 SNPs and 45 InDels identified as related to defense-response genes. Among them 112 SNPs and 12 InDels were observed in powdery mildew responsive chromosomes. Accordingly, this whole genome re-sequencing study identified SNPs and InDels associated with defense genes that will serve as candidate polymorphisms in the search for sources of resistance against powdery mildew disease and could accelerate marker-assisted breeding in melon.

## Introduction

Melon (*Cucumis melo* L.) is a highly diversified eudicot diploid species (2*n* = 2x = 24) from the Cucurbitaceae family, which includes cucumber, watermelon, and squash. Melon was previously classified into two subspecies, *C*. *melo* ssp. *agrestis* and *C*. *melo* ssp. *melo*, which were differentiated based on pubescence on the hypanthium [1] and later divided into several edible and wild varieties [2]. Melon is an important vegetable/fruit crop cultivated worldwide and highly valued for its fruit quality. More than 31 million tons of melons were produced worldwide in 2012 (Food and Agriculture Organization, http://faostat.fao.org). Formerly, Africa was designated as the primary site of origin for melons; however, current data indicate that cucumber and melon may have originated in Asian regions [3]. In recent decades, melons have emerged as an attractive model system for observable biological traits, such as fruit ripening [4], plant sex determination [5], and phloem transport [6]. A rich diversity of genomic tools have been developed for melon including a physical map [7], genetic maps [8], an EST database (http://www.icugi.org) [9], reverse genetic tools [10], BAC sequences [11], and a microarray [12].

Melon (*Cucumis melo* L.) is susceptible to various biotic and abiotic stresses [12, 13], especially powdery mildew [14, 15], which is poses a problem for Cucurbitaceous crops in the open field as well as in greenhouses. The fungi *Podosphaera xanthii* (syn. *Sphaerotheca fuliginea*) and *Golovinomyces cichoracearum* (syn. *Erysiphe cichoracearum*) are the main causal agents of powdery mildew (PM), which impedes plant growth and limits fruit quality production worldwide [15]. Powdery mildew is caused by a range of fungal races, and despite its severity on cucurbits, various accessions or cultivars show resistance to specific races [16]. However, commercial development of resistance genes against PM pathogens is hampered due to limited knowledge of melon genetic variation [17]. Analysis of a large number of races identified *Go* and *Px* resistance sources [18]; however, more information is required about the underlying genetic basis of the resistance, including the molecular underpinnings. Important resistance genes including *Go* and *Px* are listed in the Cucurbit Genetics Cooperative (cgc.ncsu.edu) for Cucurbitaceae species [19]. In addition, molecular markers based on restriction fragment length polymorphism (RFLP), random amplified polymorphic DNA (RAPD) [20], amplified fragment length polymorphism (AFLP) [21], single nucleotide polymorphism (SNP) [22] and simple sequence repeat (SSR) [23] have been implemented to study the genetic diversity of melon.

Next generation sequencing (NGS) technology has been used to extend our insights in genomic research for developing molecular markers, identification of genetic variation and gene discovery using sequencing approaches [24]. Among these technologies, whole-genome re-sequencing (WGR) has been successfully utilized to study genome diversity and discover sequence variations in crops including rice, maize, pigeon pea, chickpea, soybean, and common bean [24]. In addition to identification of genetic polymorphisms such as single nucleotide polymorphism (SNP) and insertion/deletion polymorphism (InDel), WGR permits the detection of copy number of variation (CNV) and presence/absence variation (PAV) [25]. Based on Illumina technologies, genome sequences of melon [26], watermelon [27], and cucumber [28] have been published. The melon genome was recently sequenced using the double-haploid line DHL92, derived from a cross between the melon cultivars of PI 161375 (Songwhan Charmi) and T111 (Piel de Sapo). The resulting assembled genome comprised 27,427 annotated genes with 17% of the genome being transposable elements (TEs) [26]. Considering the advantages of NGS, we have chosen four melon accessions for re-sequencing to characterize their genotypic variation in terms of SNPs, InDels, and structure variations (SVs). In addition, QTLs associated with disease resistance genes were detected for the re-sequenced variants based on available R genes from the reference genome.

## Materials and Methods

### Plant materials and disease assessment

A total of four melon (*Cucumis melo* L.) accessions, ‘SCNU1154’, ‘Edisto47’, ‘MR-1’, and ‘PMR5’, were included in this study based on PM races. Collection of PM strains, characterization and inoculation methods were followed from our published paper [29]. The foliage leaves were harvested from glasshouse-grown plants, cut into pieces and placed on MS medium in Petri dishes. For inoculation, plants were exposed to powdery mildew by conidial suspension/spraying [30] or by placement into a sedimentation tower [31]. Inoculated samples were incubated at 26°C on a 16-h light and 8-h dark cycle for two weeks. Powdery mildew disease severity was accessed using the Wilcoxon t-test with Bonferroni correction, where lesion area infection covered up to 10% (-), 10% to 30% (+), or more than 31% (++) on each sample. Additionally each melon accession was classified as resistant or susceptible using a disease index (DI) scale (0 to 5) based on the previously described calculation [32].

### DNA isolation and re-sequencing

Young leaves from each melon line were harvested from two-week-old seedlings and then subjected to genomic DNA extraction using the Qiagen DNeasy Plant Mini Kit. DNA was randomly fragmented, followed by adapter ligation and then band size-fractionated by gel electrophoresis. A sequence library of DNA of the desired length was constructed for each melon line according to the manufacturer’s instructions (Illumina, Inc., San Diego, CA). Whole-genome re-sequencing was performed using the Illumina Hiseq 2000 platform with the constructed paired-end (PE) sequence libraries at the 380Beijing Genomics Institutes (BGI)-Shenzhen (Shenzhen, China).

### Detection of SNPs and InDels

The recently released genome of the melon (*Cucumis melo L*.) accession DHL92 was downloaded from Melonomics (http://melonomics.net, _V_3.5) [26] and used as a reference genome. The sequencing data generated in this work for each melon line were examined for quality assessment using FASTX-toolkit (http://hannonlab.cshl.edu/fastx_toolkit/). The resulting clean reads from the PE sequencing for each melon accession were mapped to the melon reference genome using the Burrows-Wheeler Aligner (BWA) algorithm (v0.7.7) with default parameters [33]. Further, using SAMtools (_V_0.1.19) the resulting Binary Alignment/Map (BAM) and Sequence Alignment/Map (SAM) files were converted for sorting and indexing alignments [33]. Mark duplicates in Picard tools (_V_1.106) was used to discard duplicates, and the final sorted bam results were used for downstream analysis. In addition, detection of SNPs was carried out employing the multi-sample SNP calling approach using the Genome Analysis Toolkit (GATK, _V_3.1) [34]. This program performs local re-alignment and re-calibration to generate putative SNPs and InDels for each sample with the output format of VarScan2 (VCF). The output file consists of information related to variants and their positions along with SNP quality of coverage, mapping, genotype, and read-depth statistics [35].

### SNP and InDel annotation

From the VCF files obtained through filtering, SNPs and InDels were annotated based on genomic location and classified by the likely effects of the variations including functional categories using SnpEff (_V_4.11) [36]. The SnpEff binary database file (.bin) was generated using the melon genome annotation file (gff3) and the genome sequence. This generated database was used to annotate the effects of SNP by region, effect (high, moderate, low and modifier), and functional class (missense, nonsense, and silent) for all individuals. The generated output files, both HTML and text files, were subjected to further analysis including for localization of coding, non-coding, intronic, exonic, start codon, stop codon, upstream/downstream, splice sites, 5’ UTR and 3’ UTR regions. Then, functional enrichment analysis based on gene ontology (GO) was carried out for genes with detected SNP/InDel allelic variations. Blast2Go (https://www.blast2go.com) was also used for functional analysis of each variant; GO terms were determined with Fisher’s Exact Test and divided into three categories: biological process (BP), cellular component (CC), and molecular function (MF) [37]. Finally, detected genes were mapped to the KEGG (Kyoto Encyclopedia of Genes and Genomes) [38] database for analyzing biological pathways, and protein family annotations were assigned according to Pfam (http://pfam.xfam.org/) [39].

### Variations detected in disease resistance genes

In this study, different classes of resistance (R) genes were collected from the PRGdb database (Plant Resistance gene Database, http://prgdb.crg.eu/) [40] and taken from reports of resistance genes involved in melon disease [19]. The putative functions of genes with detected variations were determined through BLAST search using Blast2GO. This program allows the examination of putative functions of genes and characterization based on gene ontology. Further, domain analysis was carried out using the Pfam database and variant effects were correlated according to detected reads. The physical map of detected variants related to disease responses was constructed using MapChart (v2.2) [41].

### Detection of structure variations (SVs)

Abnormal orientation of paired-end reads aligned using the reference genome was analyzed using BreakDancer (v1.1.2). This program detects structural variations by clustering and comparing with previously defined SV types [42]. The structural variations of deletions (DEL), insertions (INS), inversions (INV), inter-chromosomal translocations (CTX), and intra-chromosomal translocations (ITX) were identified using the default parameters.

### Validation of SNPs in melon accessions

A total of 12 SNPs (~ 4 from each PM responsive chromosomes of 2, 5, and 12) with their corresponding 500 base pair flanking sequences were selected, and primer pairs were designed for experimental verification. The presence of each SNP was confirmed in melon accessions (SCNU1154: susceptible; Edisto, MR-1, and PMR5: resistant). The designed forward and reverse primer pairs were used for PCR amplification to verify variants. PCR amplifications were carried out using a Takara PCR Thermal Cycler with reaction mixtures (total volume of 20 μL) containing 1 × buffer, 2.0 mM MgCl_2_, 0.2 mM dNTPs, 0.2μM primers, 5 ng template DNA and 0.5 units Taq polymerase (PROMEGA, Madison, USA). The PCR cycle conditions included initial denaturation at 94°C for 5 min, then 30 cycles of denaturation at 94°C for 30 s, annealing at 59°C for 30s, extension at 72°C for 30s, and a final elongation at 72°C for 5 min. Further, PCR products were visualized using agarose gel electrophoresis and purified PCR fragments were used for sequencing (Macrogen, Korea).

### Data availability

The identified SNPs and InDels for each individual line were compared between samples for identification of unique and shared variations. The identified variations were used to plot a genome-wide chromosomal representation of structural variations using the Circos program [43]. The generated paired-end sequences were deposited into NCBI BioProject database (accession number—PRJNA300827, http://www.ncbi.nlm.nih.gov/bioproject/).

## Results and Discussion

### Disease assessment

In recent decades, a number of races have been analyzed to identify the PM inheritance for melon accessions/cultivars. Environmental factors such as temperature between 25–30°C and 98% relative humidity are requirements for sporulation of PM pathogens and severely affect the chlorotic leaves of diseased plants. The present study was carried out to confirm the PM response in four different melon accessions of SCNU1154, Edisto47, MR-1, and PMR5 by disease assessment. Young leaves were inoculated with *Podosphaera xanthii* (PM pathogen) and disease severity was measured for each line using disease index scores (0 to 5). Further confirmation of disease severity was accessed using Wilcoxon t-test with Bonferroni correction, where a visual cutoff range was fixed based on lesion area infection, 10% (-), 10% to 30% (+), and more than 31% (++) on each sample (S1 Fig). According to bioassay results on melon leaves infected with PM, ‘SCNU1154’ was highly susceptible (S, DI = 5), and Edisto47, MR-1, PMR5 lines were highly resistant (R, DI = 0). In addition, earlier work with other races also confirms that Edisto47, MR-1, PMR5 lines are highly resistant to *Podosphaera xanthii* in melon [44].

### Whole-genome sequencing

The four melon accessions ‘SCNU1154’, ‘Edisto47’, MR-1, and ‘PMR5’ were successfully sequenced using Illumina Hiseq2000 sequencing technology. Four individual paired-end DNA libraries were constructed and yielded 754,759,704 reads with an average of 15.62 million reads per accession. In total, 77.54% to 86.56% of reads were aligned on the reference melon genome. On average, each melon accession covered 82.64% of the reference genome, suggesting that our re-sequenced genomes are closely related to the reference genome. The statistics of generated raw reads, mapped alignments, and coverage of each line are summarized in Table 1. An average of 37.15% GC content was estimated for the re-sequenced samples. Mapped sequences were used for subsequent analyses.

**Table 1:** Summary of re-sequenced reads mapped onto the reference genome of melon.

Melon accession	SCNU1154	Edisto47	MR-1	PMR5
Number of reads	193,644,708	176,933,292	185,553,936	198,627,768
Number of mapped reads	167,627,400	137,194,501	149,891,959	170,252,319
Genome coverage (%)	86.56	77.54	80.78	85.71
GC %	37.04	37.70	37.15	36.71

### Detection and characterization of SNPs and InDels

**Fig 1:**
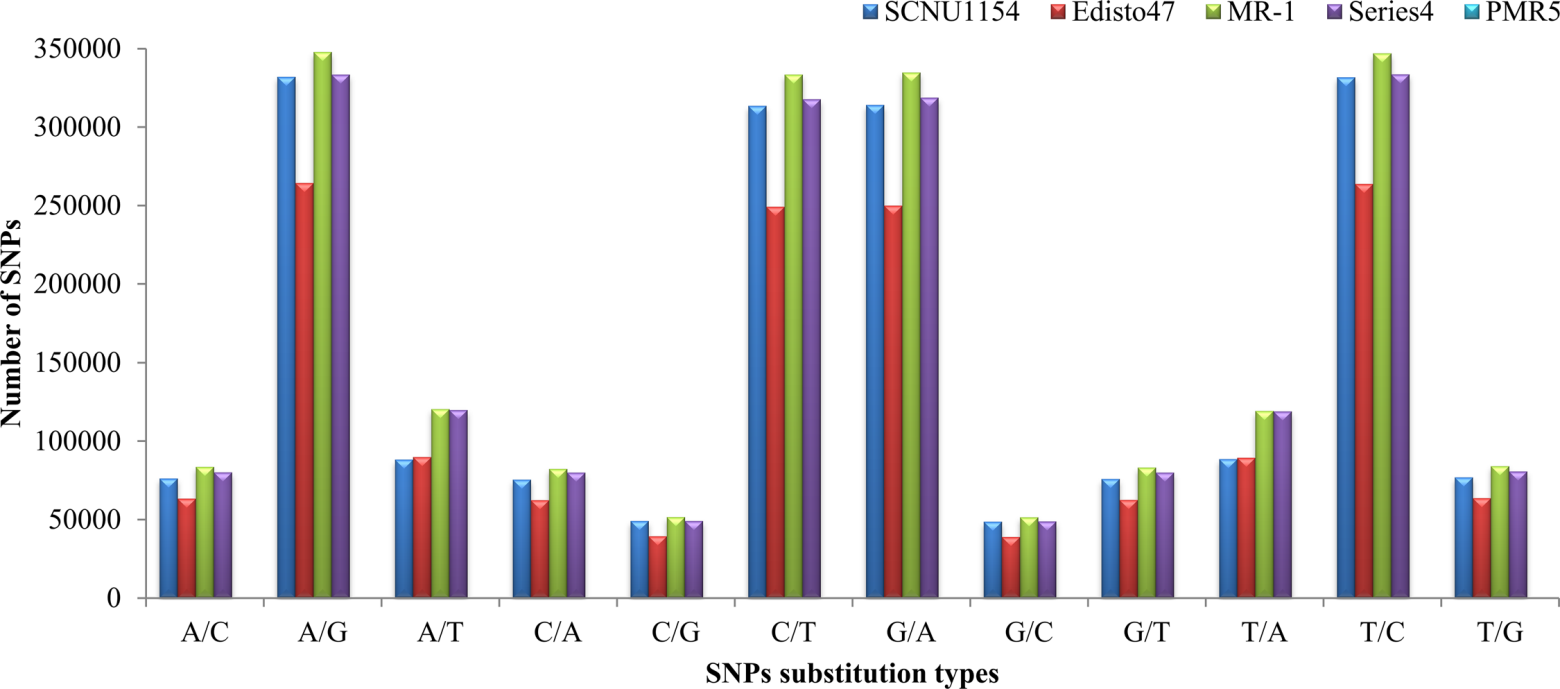
Frequency of nucleotide substitution types including transition and transversion SNPs identified in melon accessions.

Comparison to the reference genome to detect SNP variations were carried out separately for each accession using the SAMtools software. An average of 1,851,346 SNPs were detected in the accessions and the SNPs detected in ‘SCNU1154’, ‘Edisto47’, ‘MR-1’, and ‘PMR5’, respectively were shown in Table 2. These rates of SNP density in the genome correspond to approximately one change in every 217 bases (SCNU1154), 264 bases (Edisto47), 199 bases (MR-1), and 207 bases (PMR5). The identified SNPs in ‘SCNU1154’ comprised 69.02% transitions and 30.97% transversions. Among these, 6.96% were heterozygous and 93.03% were homozygous. The obtained SNPs from ‘Edisto47’ were 66.84% transitions and 33.13% transversions. Approximately 5.2% were heterozygous and 94.74% were homozygous. The SNPs of ‘MR-1’ comprised 66.85% transitions and 33.14% transversions, with approximately 5.43% heterozygous and 94.56% homozygous. The detected SNPs of ‘PMR5’ were 66.46% transitions and 33.53% transversions, with 6.62% heterozygous and 93.37% homozygous (S1 Table). In addition, transition/transversion ratio variations were observed, with average of 2.06 for all accessions (Fig 1). Furthermore, a total of 532,910, 420,691, 449,256, and 574,839 InDels were identified in ‘SCNU1154’, ‘Edisto47’, ‘MR-1’, and ‘PMR5’, respectively, by comparing their individual genome sequences to the reference genome (Table 2). Among these detected InDels, ‘SCNU1154’ had 178,041 insertions and 354,862 deletions; ‘Edisto47’ had 131,313 insertions and 289,374 deletions; ‘MR-1’ had 175,534 insertions and 273,722 deletions; and ‘PMR5’ had 175,891 insertions and 398,944 deletions (S2 Fig). The approximate InDel density variant rate observed for each accession was one change in every 763 bases (SCNU1154), 967 bases (Edisto47), 905 bases (MR-1), and 707 bases (PMR5).

**Table 2:** Distribution of detected variants in each melon accession.

Chromosome	SNPs				InDels			
	SCNU1154	Edisto47	MR-1	PMR5	SCNU1154	Edisto47	MR-1	PMR5
**Pseudo Chr.0**	78,691	52,931	71,759	74,246	12,959	8,205	11,595	12,429
**Pseudo Chr.1**	117,470	97,749	163,316	119,765	106,464	97,594	38,420	171,064
**Pseudo Chr.2**	96,546	87,604	147,132	122,491	28,402	22,428	31,105	29,991
**Pseudo Chr.3**	189,676	153,206	159,963	186,712	37,636	27,071	33,312	35,595
**Pseudo Chr.4**	220,731	172,321	208,904	215,031	48,527	35,461	43,426	46,701
**Pseudo Chr.5**	129,898	132,998	139,763	152,987	32,987	26,929	32,134	33,859
**Pseudo Chr.6**	126,417	111,452	192,139	126,829	38,956	30,424	41,376	37,438
**Pseudo Chr.7**	177,919	145,971	159,569	173,281	53,396	44,152	49,938	36,092
**Pseudo Chr.8**	125,771	115,266	168,185	133,293	35,977	28,177	37,800	35,394
**Pseudo Chr.9**	85,698	73,609	125,560	93,311	27,613	21,068	29,762	27,125
**Pseudo Chr.10**	148,306	138,205	132,878	169,368	29,798	23,375	27,482	30,122
**Pseudo Chr.11**	230,617	135,058	210,387	239,101	45,480	30,490	40,118	45,547
**Pseudo Chr.12**	142,255	120,015	158,783	154,249	34,715	25,317	32,788	33,482
**Total**	**1,869,995**	**1,536,385**	**2,038,338**	**1,960,664**	**532,910**	**420,691**	**449,256**	**574,839**

### Chromosomal distribution of SNPs and InDels

**Fig 2:**
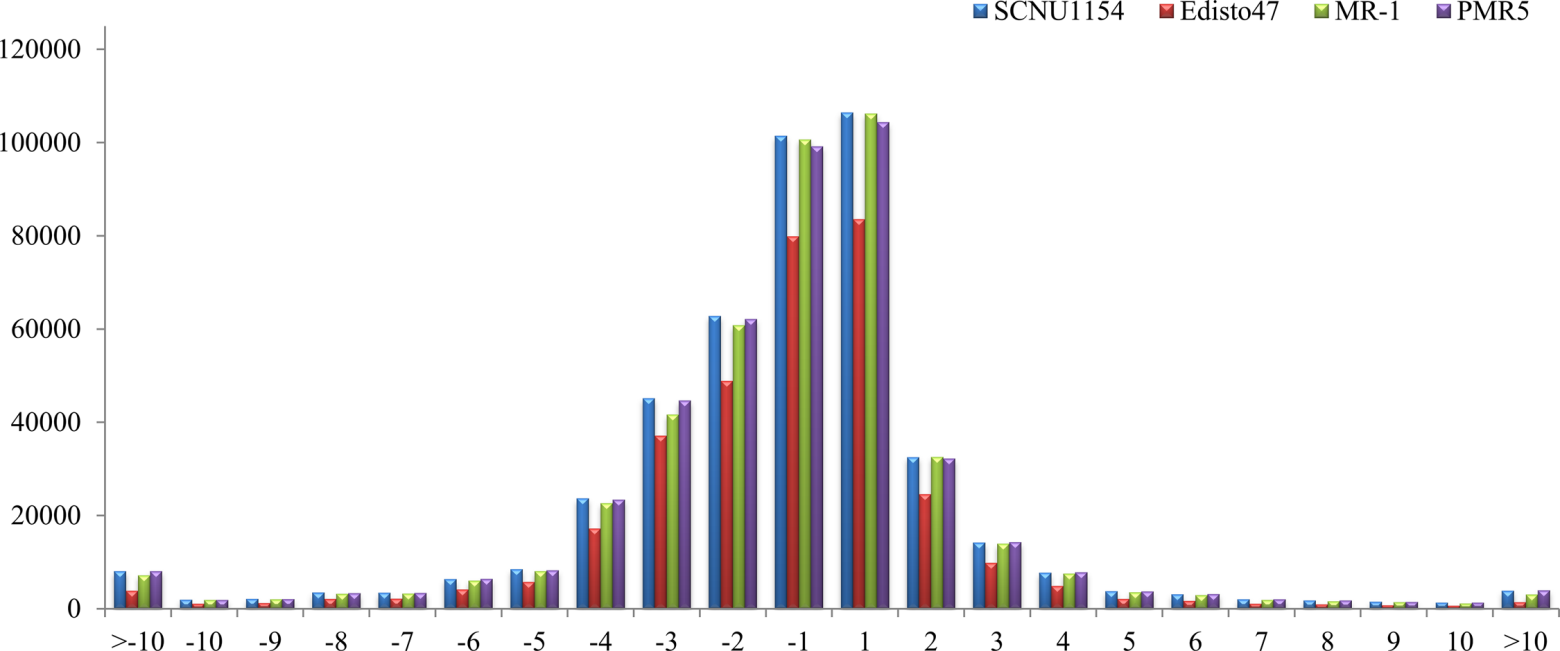
InDel length distribution in melon accessions.

**Fig 3:**
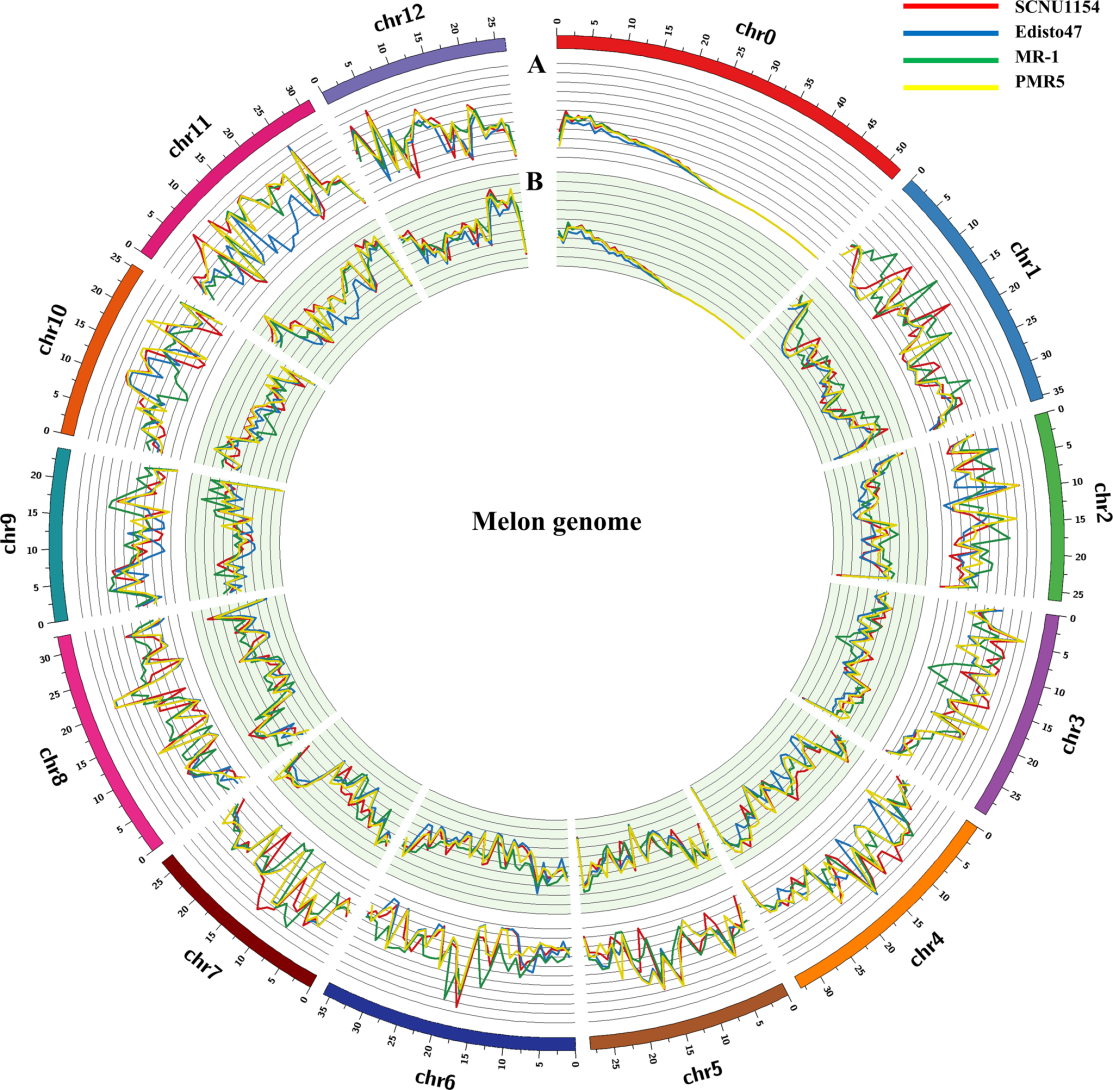
Circos plot of the detected genetic variations in the melon genome. Distribution of (A) SNPs and (B) InDels are represented in each melon pseudo-chromosome.

The reference genome of melon, including Pseudo chromosomes 1 to 12 (Chr.1 to 12) as well as non-anchored scaffolds and contigs (Chr.0) (hereafter pseudo chromosomes are referred to as chromosomes in this manuscript) were used for analyzing chromosomal distributions of the identified SNPs. The SNPs in all accessions were widely distributed to each chromosome including non-anchored scaffolds and contigs (S3A Fig). Chr.0 contained the fewest SNPs, with an average of 58,117 among all accessions. SNP coverage in ‘SCNU1154’ was lowest in Chr.2 and Chr.9 compared to other chromosomes. Similarly, Chr. 1, 2, and 9 from ‘Edisto47’ and Chr. 9 from ‘PMR5’ had fewer SNPs compared to other chromosomes within the respective accessions. The ‘MR-1’ SNPs were distributed among all chromosomes. The length of InDels (insertions and deletions) ranged between 1 to 22 bp according to accession (Fig 2). In this study, the largest InDel size (22 bp, 3 occurrences) was detected in ‘PMR5’ and most of the InDels were in less than 10 bp. In addition, InDel lengths varied between the accessions (S2 Table). Further, a high rate of single bp InDels existed in all accessions among the detected InDels. The distributions of identified InDels in the melon chromosomes is shown in S3B Fig. Comparing all accessions, the highest abundance of InDels was detected in Chr.1 and a small proportion of InDels was detected in Chr.0 (11,297 avg.). The remaining chromosomes showed similar patterns of InDel distribution according to total detected InDels per accession. The short InDels (1–5 base pairs) may have deleterious effects on gene functionality during expression or transcriptional processes [25]. Overall, the maximum number of detected SNPs and InDels was in ‘PMR5’, with relatively low variation observed in ‘Edisto47’. The frequency of SNPs was highest in ‘MR-1’ in comparison to the other accessions, while the most InDels were detected in ‘PMR5’. The detected SNPs and InDels from each accession were visualized in the melon chromosomes using Circos (Fig 3).

### Annotation of SNPs and InDels

To annotate the detected SNPs and InDels from SAMtools, sequence information and annotation files of the melon genome were used to evaluate the possible effects of the variants in genomic, exonic, and functional categories. Approximately 46.5%, 42.7%, 43.7%, and 44.8% SNPs were in intergenic regions, 5.7%, 6.7%, 6.2%, and 6% were intronic, and 1.7%, 1.7%, 1.6%, and 1.6% exonic in SCNU1154’, ‘Edisto47’, ‘MR-1’ and ‘PMR5 accessions, respectively. The effects of SNP variations were classified into four classes, “high effect”, “moderate effect”, “low effect”, and “modifier”, according to SnpEff [36]. In all, 98% (Avg.) of SNPs were classified as sequence modifiers (introns or affecting non-coding genes) and moderate effect accounted for 0.81% of the SNPs on average. The low effect variants ranged from 0.95 to 1.08% according to accession. The remaining high effect variants ranged from 950 to 1,192 SNPs (0.02% avg.) within the tested accessions. S3 Table describes the proportion of variants in each effect category.

**Fig 4:**
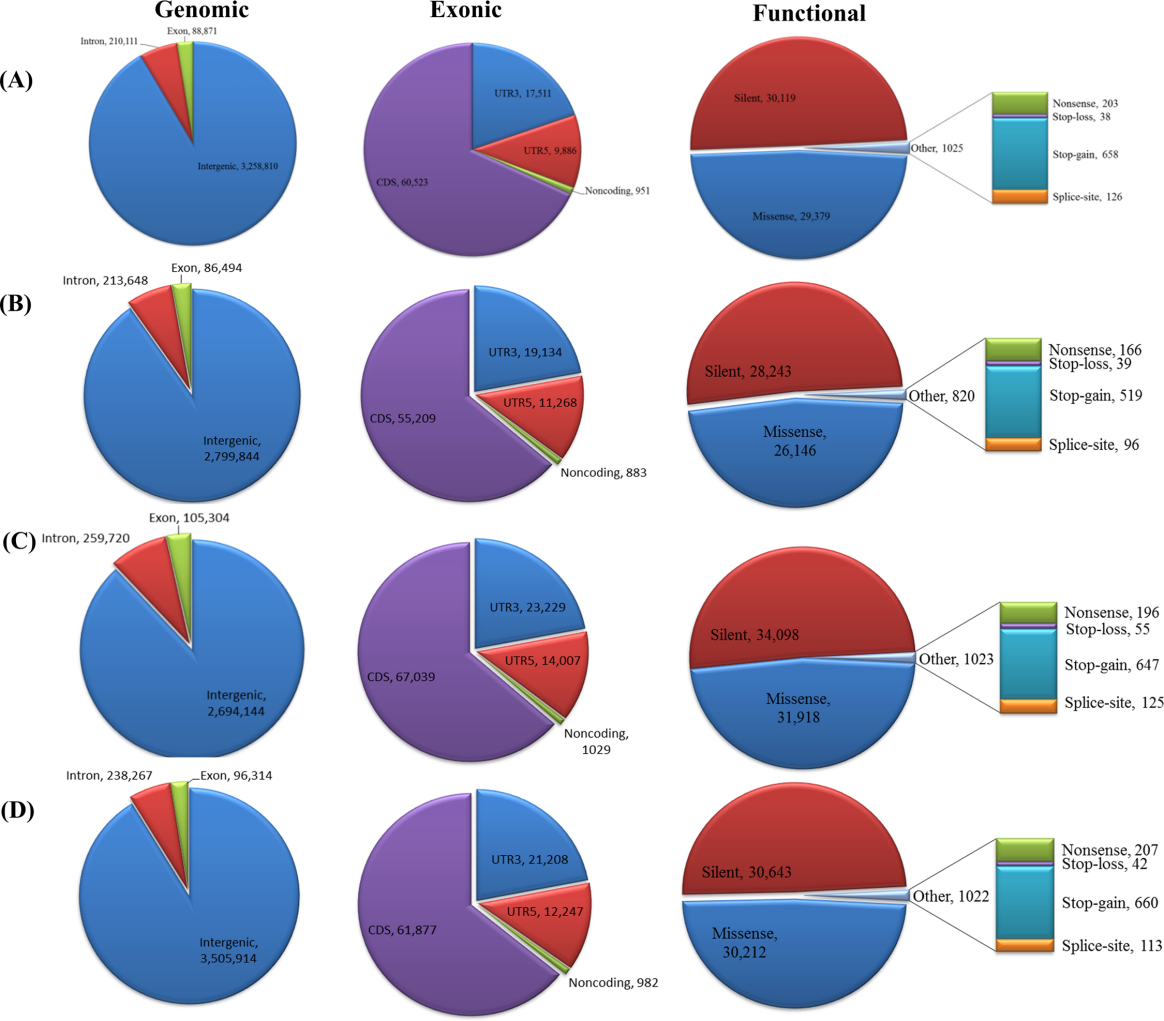
Genetic variation of SNPs detected in (A) SCNU1154, (B) Edisto47, (C) MR-1, and (D) PMR5.

Functional classes of missense, nonsense, and silent variants were evaluated for the detected SNPs. A total of 1,192 SNPs (0.034% of total SNPs) were considered as high effect variants in ‘SCNU1154’ and included 49.05% missense SNPs and 49.87% silent SNPs, with a 0.98 missense/silent ratio. In ‘Edisto47’, 950 SNPs were assigned as high effect (0.031% of total SNPs) and comprised 47.8% missense SNPs and51.26% silent SNPs, with a missense/silent ratio of 0.93. In the case of ‘MR-1’, a total of 1,176 SNPs (0.029% of total SNPs) were measured as high effect variants, with 48.049% missense SNPs and 50.99% silent SNPs and a 0.94 missense/silent ratio. The ‘PMR5’ accession exhibited 1,186 SNPs (0.031% of total SNPs) in the high effect variation category; 49.30% were missense and 49.63% were silent. In addition, small proportions of nonsense SNPs were detected in SCNU1154’,’Edisto47’,’MR-1’and ‘PMR5 accessions (1.07%, 0.93%, 0.96%, and 1.06%, respectively; Fig 4).

**Fig 5:**
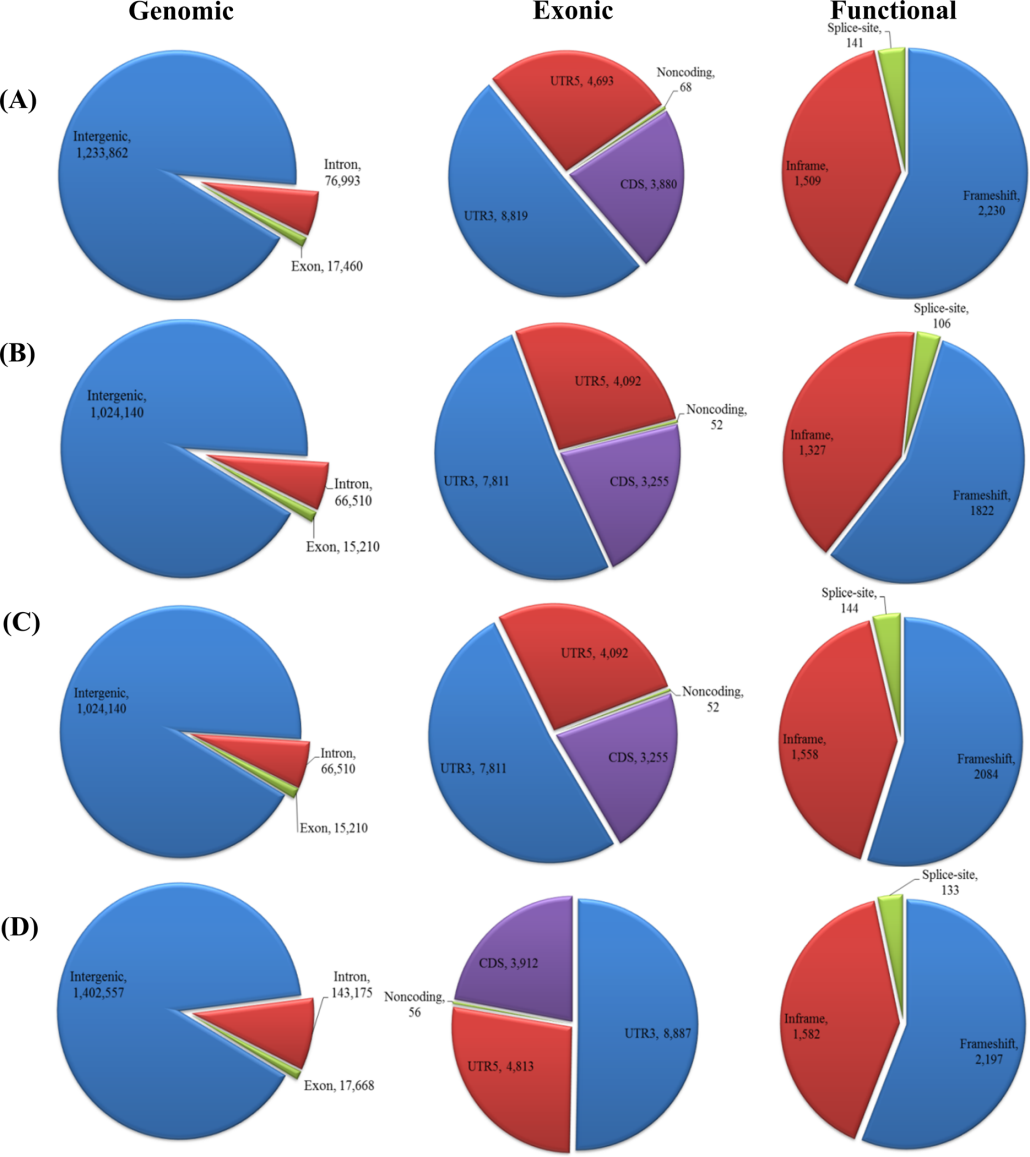
Genetic variation of InDels detected in (A) SCNU1154, (B) Edisto47, (C) MR-1, and (D) PMR5.

Similar methodology was used to annotate the detected InDels based on the melon reference genome using SnpEff. Among total InDels, 35.1%, 32.9%, 39.2%, and 28.3% were in intergenic regions, 5.6%, 5.8%, 7.8%, and 9% were intronic, and 0.2%, 0.2%, 0.3%, 0.2% were exonic in ‘SCNU1154’, ‘Edisto47’, ‘MR-1’, and ‘PMR5’, respectively. An average of 99.5% InDels (among all InDels from all accessions) was classified as sequence modifiers and 0.12% as moderate effect. The low effect variants were observed in the range from 0.09 to 1.15% according to accession. The remaining high effect variants accounted for 0.19% in total (S3 Table). The percentages of high-effect InDels (SCNU1154: 0.18%, Edisto47: 0.18%, MR-1: 0.23% and PMR5: 0.15%) were fairly small. In addition, possible functional roles such as frameshift, in-frame, and splice-site variants were assessed for all detected InDels (Fig 5). The observed InDels with high effect variants were higher than for SNPs, likely because InDels may cause frameshift effects in the sequence [45].

### Comparative analysis and annotation of variant genes

**Fig 6:**
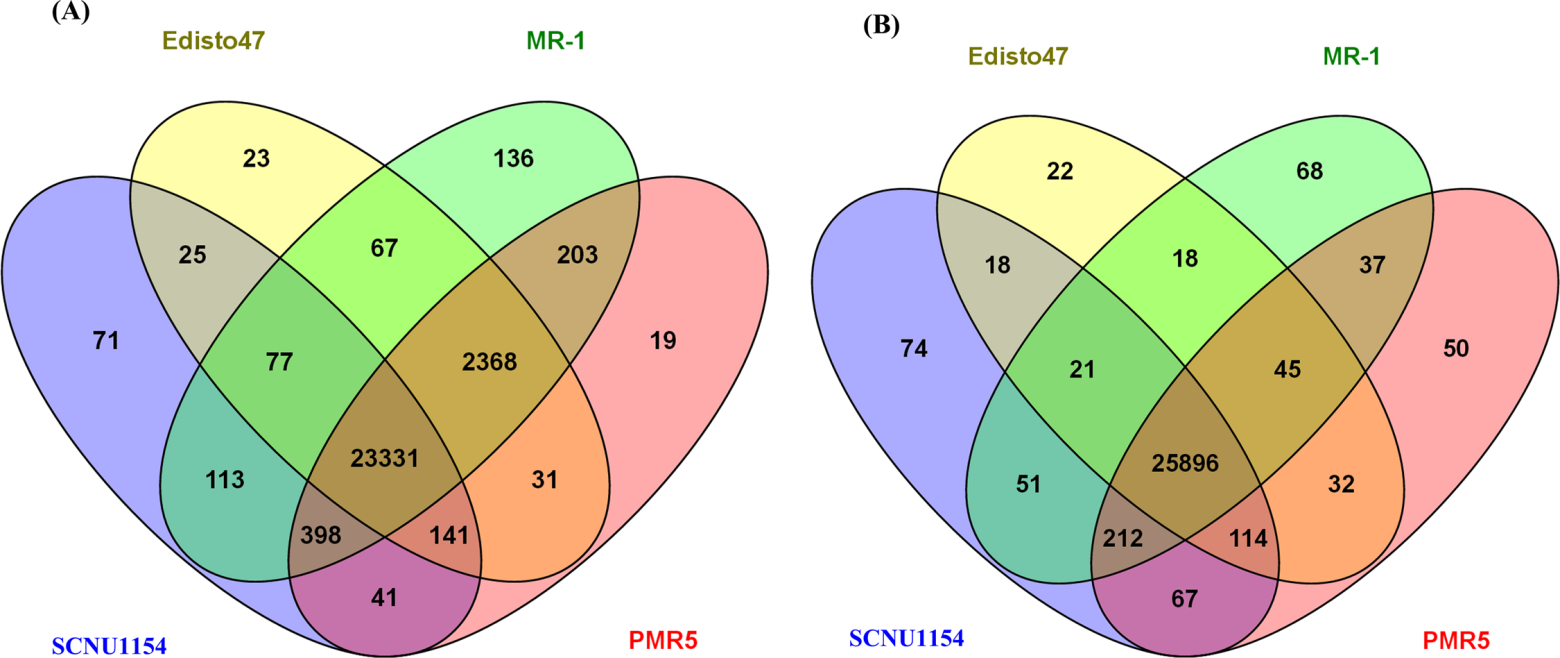
Comparison of (A) SNP and (B) InDel variations detected in each melon accession.

Detected SNPs and InDels were examined to identify non-redundant genes in each accession. This analysis revealed that 24,197 genes with detected SNPs and 26,453 genes with detected InDels were in ‘SCNU1154’, 26,063 genes with detected SNPs and 26,166 genes with detected InDels were in ‘Edisto47’, 26,693 genes with detected SNPs and 26,348 genes with detected InDels were in ‘MR-1’, and 26,532 genes with detected SNPs and 26,453 genes with detected InDels were in ‘PMR5’. These genes were compared to each other to identify the unique and common genes containing SNP and InDel variations between all accessions (Fig 6). This comparative result revealed the presence of 71 unique SNP-containing genes in ‘SCNU1154’, possessing 1,371 SNPs. Likewise, 23, 136, and 19 unique genes with 823, 19,931, and 1,298 SNPs were found in ‘Edisto47’, ‘MR-1’, and ‘PMR5’, respectively. A total of 74, 22, 68, and 50 unique genes were identified with 613, 156, 1,438, and 1,298 InDel variations in ‘SCNU1154, ‘Edisto47’, ‘MR-1’, and ‘PMR-5’ accessions, respectively. Notably, 23,331 unique SNP genes and 25,896 unique InDel genes were identified as common to all four accessions.

We used the Blast2GO program for functional enrichment analysis of detected high and moderate effect variants (SNPs and InDels) in all accessions. The closely related species, *Cucumis sativus* was used to obtain the putative functions using BLAST searches. Based on functional GO annotations of SNP-containing genes, the assigned GO terms belonging to the biological process class ranked the highest (avg. of 6,646,37% and 11, 3295,40%), followed by the molecular function class (avg. 5,845,32% and 85,473,30%), and the cellular component class (avg. 5,636,31% and 83,929,30%) for high- and moderate-effect variants, respectively. The GO terms obtained for InDel genes revealed that an average of 19,310 and 11,654 (40% and 39%) were in biological process, followed by cellular component with 14,423 and 11,654 (40% and 31%), and molecular function with 14,423 and 9,177 (30%, and 30%), respectively. Of note, ‘catalytic activity’ (GO: 0003824) and ‘binding’ (GO: 0005488) were highly represented GO terms in the molecular function category. Under the classification of biological process ‘cellular process’ (GO: 0009987) and ‘metabolic process’ (GO: 0008152) were significantly represented among GO terms. ‘Cell part’ (GO: 0044464) and ‘cell’ (GO: 0005623) are most abundant GO terms in cellular component (S4 and S5 Figs).

### Structural Variants (SVs)

Variations such as deletions (DEL), insertions (INS), inversions (INV), inter-chromosomal translocations (CTX), and intra-chromosomal translocations (ITX) are defined as genomic structural variations if they consist of greater than 1 kb sequence rearrangement [46]. In the present study, SVs were detected for each melon accession by making use of the BreakDancer program with default parameters. We found a total of 47,743 (SCNU1154), 44,510 (Edisto47), 40,908 (MR-1), and 49,237 (PMR5) SVs with an average of 45.5% variation when compared to the reference genome (Table 3). For these SVs, InDels of deletion events (average = 45%) outnumbered insertions (average = 11%). The number of SVs found in this study was consistent with those found in other plant species [46]. The other variations including inversions (INV), inter-chromosomal translocations (CTX), and intra-chromosomal translocations (ITX) accounted for an average of 11%, 27.25% and 6.5%, respectively. In brief, SVs of individual melon accessions ‘SCNU1154’, ‘ Edisto47’, ‘MR-1’, and ‘PMR5’ were 53%, 58%, 56%, and 53% InDels, 12%, 10%, 11%, and 11% INV, 7%, 6%, 7%, and 6% ITX, and 28%, 26%, 26%, and 29% CTX, respectively. In addition, a total of 26,450 InDel, 5,339 INV, 14,234 CTX, and 3,214 ITX SVs detected in Chr.0, and CTX events were not detected in Chr.12 for any of the accessions (S4 Table).

**Table 3:** Chromosomal distribution of structural variants detected for each accession.

Chromosomes	SCNU1154	Edisto47	MR-1	PMR5
**Pseudo Chr.0**	17,868	12,441	13,512	17,207
**Pseudo Chr.1**	2,882	2,925	2,724	2,970
**Pseudo Chr.2**	2,987	3,933	3,073	3,437
**Pseudo Chr.3**	2,648	2,675	2,199	2,803
**Pseudo Chr.4**	3,340	3,595	2,963	3,567
**Pseudo Chr.5**	2,194	2,335	1,915	2,442
**Pseudo Chr.6**	2,949	3,585	3,111	3,231
**Pseudo Chr.7**	2,411	2,574	2,063	2,566
**Pseudo Chr.8**	2,109	2,088	2,030	2,164
**Pseudo Chr.9**	2,011	2,243	1,937	2,124
**Pseudo Chr.10**	1,658	1,769	1,369	1,775
**Pseudo Chr.11**	2,625	2,225	2,131	2,755
**Pseudo Chr.12**	2,061	2,122	1,881	2,196
**Total**	**47,743**	**44,510**	**40,908**	**49,237**

### Variants in resistance genes

The defense responses genes related to disease resistance were identified through functional annotations of detected SNPs and InDels compared to the reference genome. Previously reported disease resistant genes/QTLs from PRGdb in *Cucumis melo* were taken into consideration. A total of 411 putative disease resistance genes have been reported in melon, and this number is low compared to the numbers of resistance genes known in *Arabidopsis* (526), grape (690), and rice (1206) [26]. In melon, resistance genes have been identified for diseases including zucchini yellow mosaic virus (ZYMV) (*Zym*; [47]), *watermelon mosaic virus-Morocco* (Potyvirus) (*Nm*; [48]), aphis gossypii (*Fn*; [49]), papaya ring spot virus- watermelon type (PRSV) (*Prv*; [47, 50]), cucurbit aphid borne yellows viru*s* (CABYV) (*cab-1* and *cab-2*; [51]), beet pseudo yellows virus (BPYV; [52]), *cucurbit yellow stunting disorder virus* (CYSDV; [53]), lettuce infectious yellows virus (LIYV; [54]), *cucurbit leaf crumple virus* (CuLCrV; [55]), *melon necrotic spot virus* (MNSV; [56]), cucumber green mottle mosaic virus (CGMMV) (*cgmmv-1* and *cgmmv-2*; [57]), and fusarium wilt (*Fom-1*; [58], *Fom-2*; [59, 60]). In addition, several *powdery mildew resistance* (*Pm*) genes have been mapped or cloned in the melon genome (*Pm-1*; [61], *Pm-2*; [62], *Pm-3*; [63], *Pm-4* and *Pm-5*; [64], *Pm-6*; [65], *Pm-8*; [66], and *Pm-F* & *Pm-G*; [67]). In addition, several studies were reported that *Mildew Locus O* (MLO) genes act as susceptibility factors in PM disease and their inactivation of specific MLO genes (knock-out or knock-down) leads to mediating form of *mlo* resistance [68].

**Fig 7:**
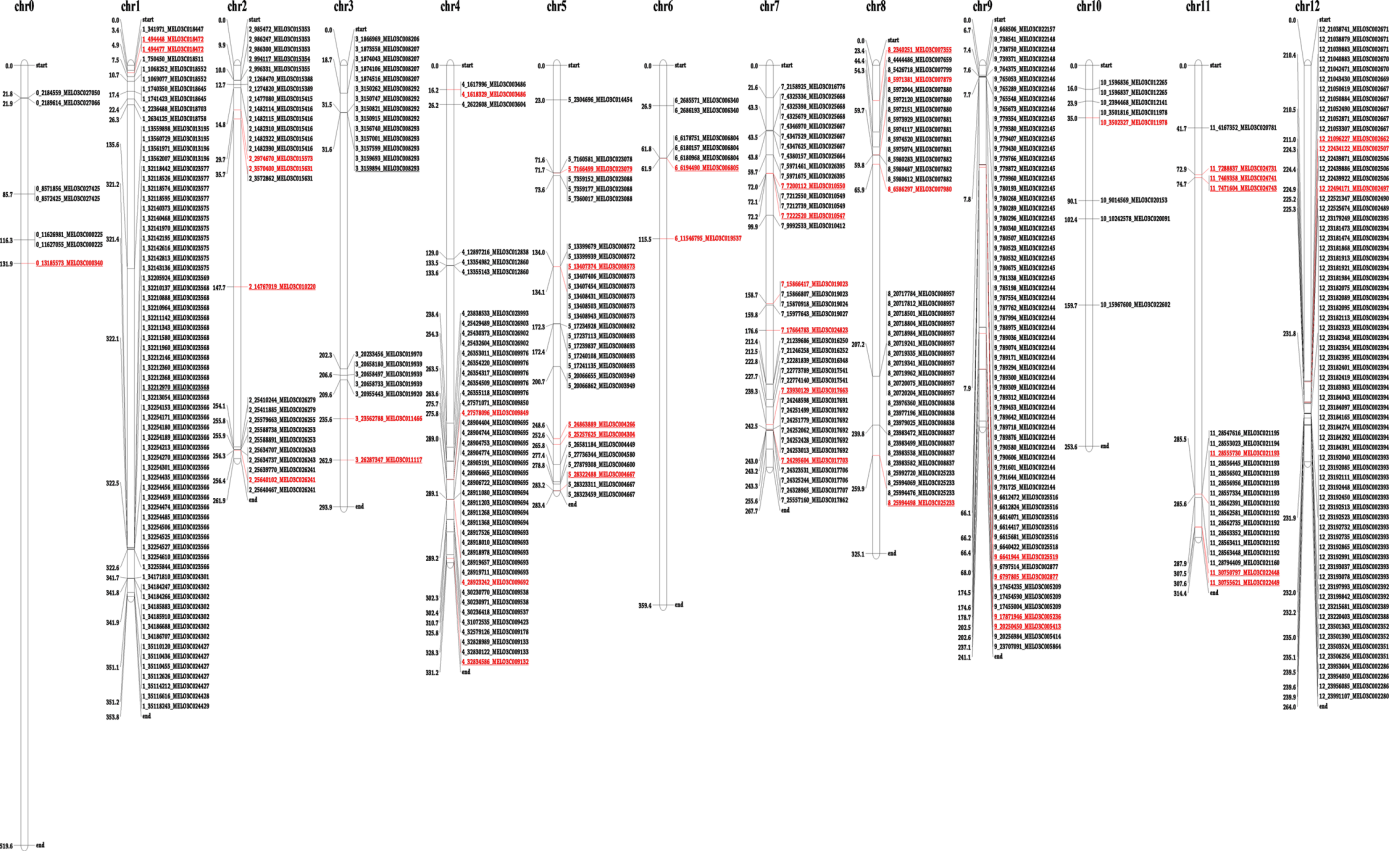
Physical map of detected SNPs and InDels related to defense response genes. A total of 390 SNPs and 45 InDels are represented with physical map position. The different colors represent moderate-effect SNPs (black), high-effect SNPs (black with underline), moderate-effect InDels (red), and high-effect InDels (red with underline).

In this study, comprehensive domain analyses of encoded defense genes were performed. A total of 11,867 SNPs were identified in 585 defense-related genes in all four accessions and found to be distributed across all chromosomes. Among them, different classes of disease resistance-related protein domains were found, including CNL [CC-NB-LRR, proteins containing coiled-coil (CC), nucleotide-binding site (NBS), and leucine-rich repeat (LRR) domains], TNL [TIR-NB-LRR, Toll-interleukin receptor-like, nucleotide binding site (NB) and LRR domains], RLP (receptor serine/threonine kinase-like domain), and an extracellular LRR (ser/thr-LRR); in addition, other kinase domains with an extracellular LRR (RLK) were detected in all four accessions. The details of defense-related genes with detected SNPs are tabulated in S5 Table. Among 11,867 SNPs in defense-related genes, 4 SNPs predicted to be high effect variants (affecting splice site regions, start and stop codons) and 386 predicted as moderate effect variants (non-synonymous coding, codon insertions and deletions) were found within 139 defense-related genes. In addition, functional enrichment analysis showed that GO terms of catalytic activity (GO: 0003824), and binding (GO: 0005488) were highly represented in the molecular function category for these identified disease-related genes. Of note, these domains are commonly involved in plant defense mechanisms and mediate disease resistance in plants [40]. Similar to SNPs, 3,429 InDels were identified with 31 InDels (30 genes) and 14 InDels predicted to have high and moderate effects, respectively (S6 Table). Using the advances of NGS technology in melon re-sequencing, the presence and absence of defense-related genes has been demonstrated [69]. Leida et al [70] re-sequenced 175 melon accessions to identify the variability of SNPs and characterized sugar accumulation and fruit ripening-related genes. However, there has been no attempt reported for the development of QTLs associated with disease resistance-related genes in melon, especially for powdery mildew disease assessment using re-sequencing. Accordingly, we constructed a disease-associated QTL map for 390 SNPs and 45 InDels identified from this study as related to defense-response genes (Fig 7).

Recent evidences reveled that MLO gene specific knock-out or knock-down leads to PM resistant in arabidopsis, tomato, pea, pepper, grapevine and wheat [68]. For this study we have collected 14 MLO genes information from our published paper [71]. Further, the genes were characterized using basic bioinformatics analysis for identification of gene position, locus, and lengths. Then these informations were correlated with the present study and investigated for their SNPs and InDels variation. The comparative analysis of the MLO genes revealed 1,123, 1,123, 1,615, and 1,663 SNPs and 486, 386, 411, 464 InDels in ‘SCNU1154, ‘Edisto47’, ‘MR-1’, and ‘PMR-5’ accessions, respectively. Notably, 438 SNPs and 125 InDels were identified as common to all four accessions (S6 Fig and S8 Table). We hope that these MLO genes associated SNPs and InDels would help to accelerate the development of disease resistant marker against PM.

### SNP validation in powdery mildew responsive chromosomes

In melon, several PM resistant genes and resistant trait QTL have been reported in chromosome 2, 5, and 12 [72]. We found 23, 24, 65 SNPs and 4, 5, 3 InDels from PM responsive chromosomes of 2, 5, and 12 respectively. Further, we conducted experimental validation for several detected SNPs related to possible disease resistance genes using 12 primer pairs (S7 Table) targeting of 12 SNPs from PM responsive chromosomes. During the process of retrieving the sequence for SNP selection we noticed poly N repeats those SNPs were omitted from further analysis. This inconsistency is due to the incompleteness of draft reference genome [73]. The selected SNPs were confirmed by their amplification by using four melon accessions of SCNU1154 (susceptible), Edisto (resistant), MR-1(resistant), and PMR5 (resistant). The SNPs were amplified by flanking primers in all four re-sequenced melon lines. In addition, MELO3C015353 and MELO3C015354 gene from chr.2 has been reported for PM candidate resistant gene in Edisto47 [72]. In this study, we observed a total of 4 SNPs in MELO3C015353 and MELO3C015354 genes from re-sequenced melon lines (S5 Table). MELO3C015354 is a CNL class of gene (CC-NB-LRR domains) and MELO3C015353 is a NL class gene lacking CC domain. The SNP (nucleotide transition of A to C—Chr.2: 994117) observed in MELO3C015354 gene caused a stop lost/splice region variant. Three missense non-synonymous variant was observed in MELO3C015353 gene (Chr.2: 985472, 986247, and 986300). Further, melon accession/allele-specific marker development are in progress form detected SNPs with associated genes. The identified SNPs associated with PM disease resistant QTL from re-sequenced melon lines may provide novel insights to improve the disease resistance in melon accessions by using advanced molecular technologies.

## Conclusions

The importance of next generation technology is rapidly increasing in plant research by offering a variety of applications related to understanding genetic variation and facilitating marker identification/characterization in different plant varieties. In this present study, we have conducted PM disease assessment for ‘SCNU1154’, ‘Edisto47’, ‘MR-1’, and ‘PMR5’ accessions of melon and conducted whole-genome re-sequencing for each accession. Our large-scale cataloging of variations found among all accessions will be useful for marker development including SNP array design and fine mapping. In addition, the reference genome used in this study has gaps and uncertainty in the few regions of sequences. Particularly González VM et.al reported a 1-Mb region of NBS-LRR genes (MELO3C004235 to MELO3C004331) re-annotated in chromosome 5 of melon genome (improved sequence 5,190,204–6,256,576 from CM 3.5: 5,190,204–6,256,576) [73]. This variations should be considered in future to improve marker development. Further, our reported R gene-related markers especially for PM disease associated genes will be suitable for design genotyping by sequencing (GBS), linkage map construction, and development of PM disease-associated QTLs/genes. In addition, our suggested makers from this study provide support for marker-assisted breeding in melon and highlight areas for further study to understand the functional significance of each accession.

## Supporting Information

S1 Fig Response of melon accessions to powdery mildew (*Podosphaera xanthii*). Shown are the leaf disc tests after inculcation (A) and measured infected areas (B) of young leaves with representative infection severity.
(TIF)

S2 Fig Distribution of the number of InDels identified in melon accessions. (TIF)

S3 Fig Chromosomal distribution of detected (A) SNP and (B) InDel variations in melon pseudo-chromosomes.
(TIF)

S4 Fig GO classification of detected high- and moderate-effect SNPs in (A) SCNU1154, (B) Edisto47, (C) MR-1, and (D) PMR5 accessions.
(TIF)

S5 Fig GO classification of detected high- and moderate-effect InDels in (A) SCNU1154, (B) Edisto47, (C) MR-1, and (D) PMR5 accessions.
(TIF)

S6 Fig Comparative analysis of (A) SNP and (B) InDel variants in MLO genes.
(TIF)

S1 Table Frequency of transitions and transversions in each accession. (XLSX)

S2 Table The distribution of InDel lengths in each accession. (XLSX)

S3 Table Distribution of SNP/InDel effects by type and region across melon accessions. (XLSX)

S4 Table Chromosomal distribution of detected structure variations. (XLSX)

S5 Table SNP variations in R genes. (XLSX)

S6 Table InDel variations in R genes. (XLSX)

S7 Table Primers used in this study. (XLSX)

S8 Table SNP and InDel variations in MLO genes. (XLSX)
