# Supplementary figures and images for: Whole Genome Re-Sequencing and Characterization of Powdery Mildew Disease-Associated Allelic Variation in Melon
Source: PLoS One. 2016 Jun 16;11(6):e0157524. doi: 10.1371/journal.pone.0157524 (PMC4911151; doi:10.1371/journal.pone.0157524)

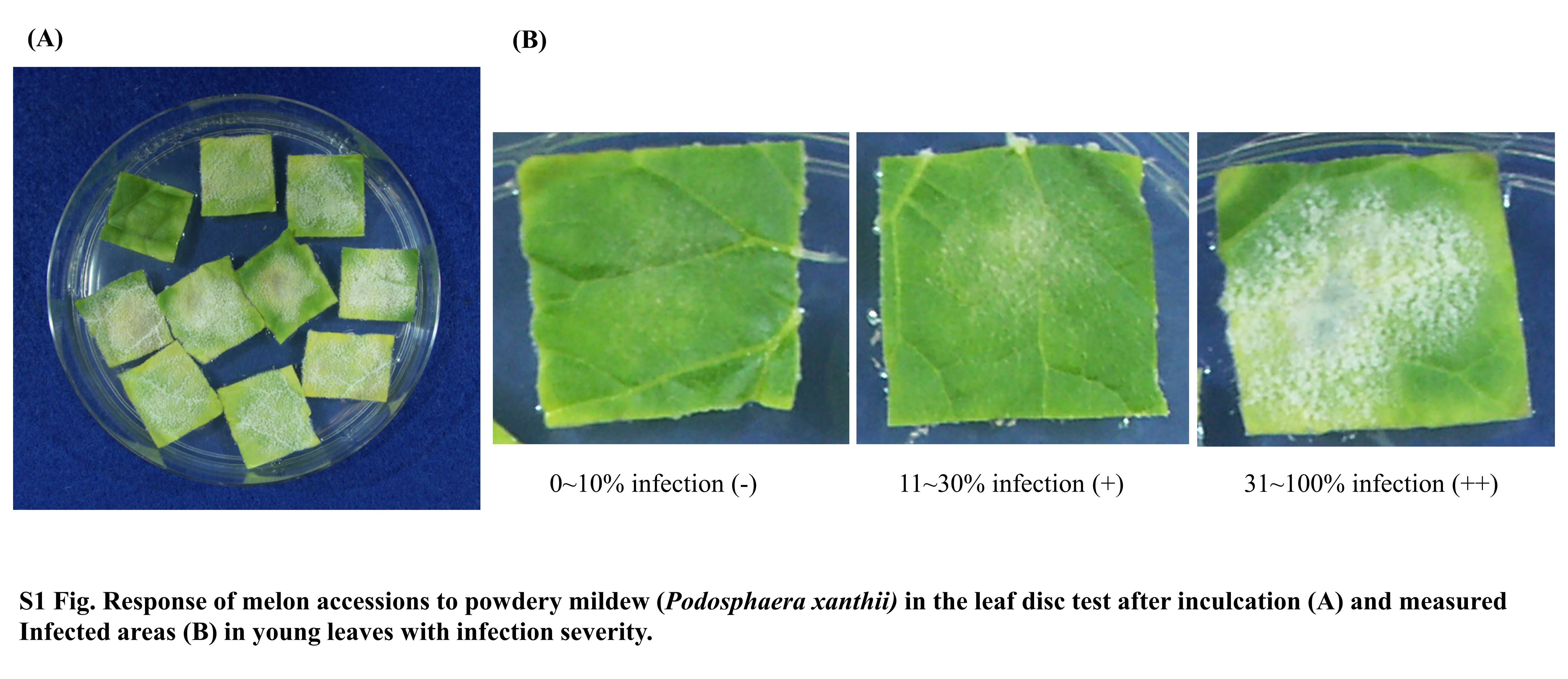

Supplement: S1 Fig — Shown are the leaf disc tests after inculcation (A) and measured infected areas (B) of young leaves with representative infection severity. (TIF) [file pone.0157524.s001.tif]

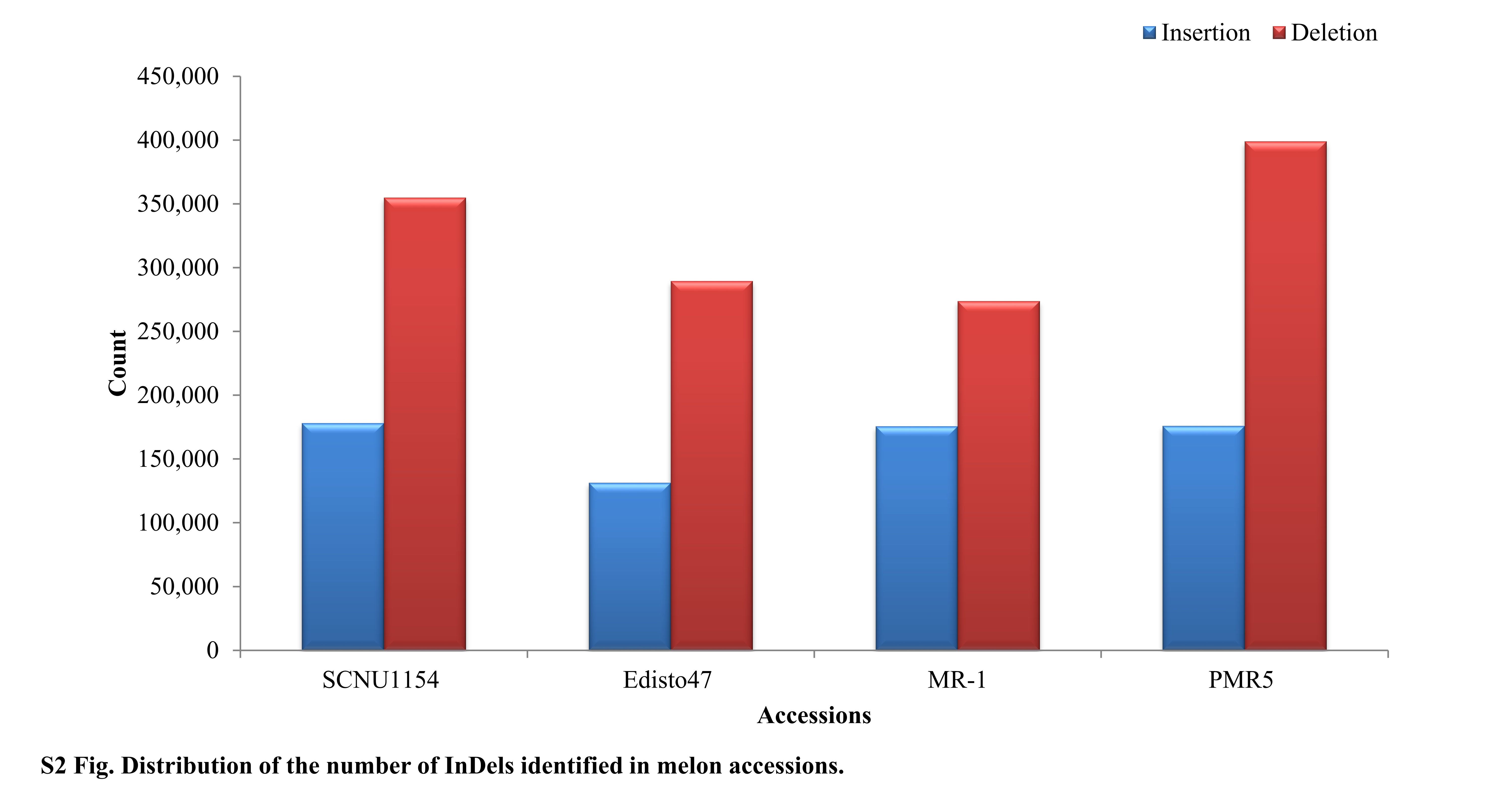

Supplement: S2 Fig — (TIF) [file pone.0157524.s002.tif]

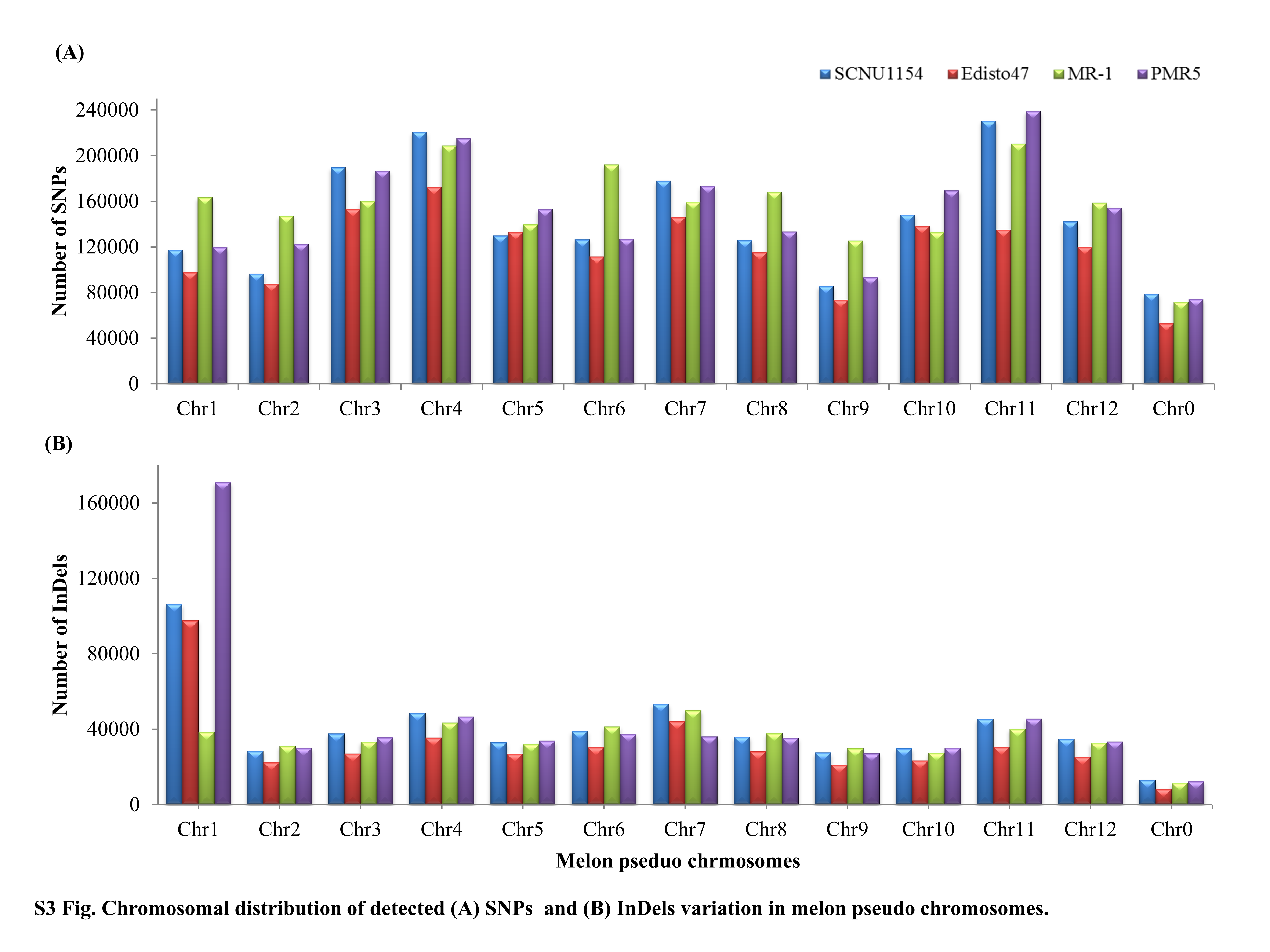

Supplement: S3 Fig — Chromosomal distribution of detected (A) SNP and (B) InDel variations in melon pseudo-chromosomes. (TIF) [file pone.0157524.s003.tif]

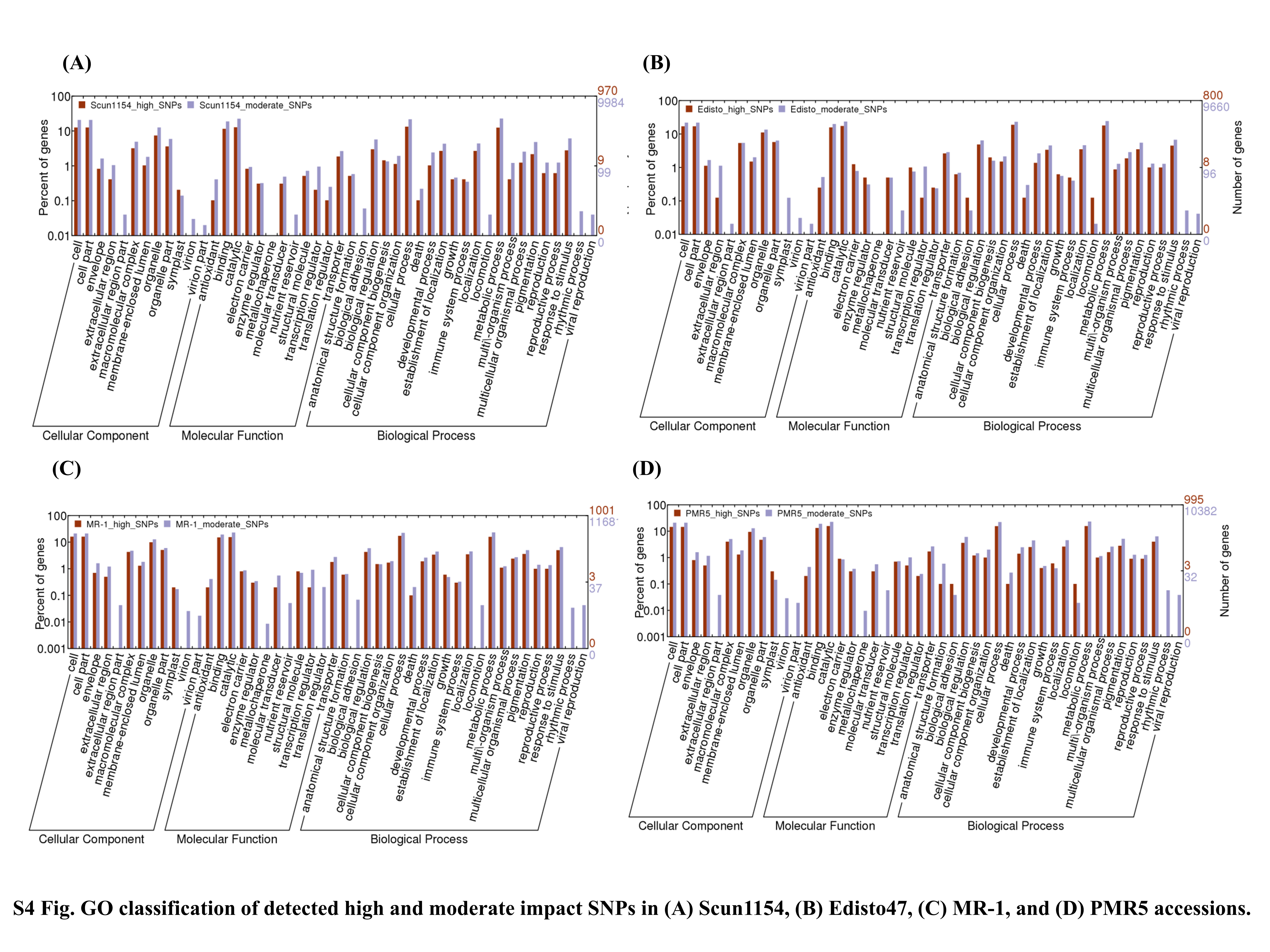

Supplement: S4 Fig — GO classification of detected high- and moderate-effect SNPs in (A) SCNU1154, (B) Edisto47, (C) MR-1, and (D) PMR5 accessions. (TIF) [file pone.0157524.s004.tif]

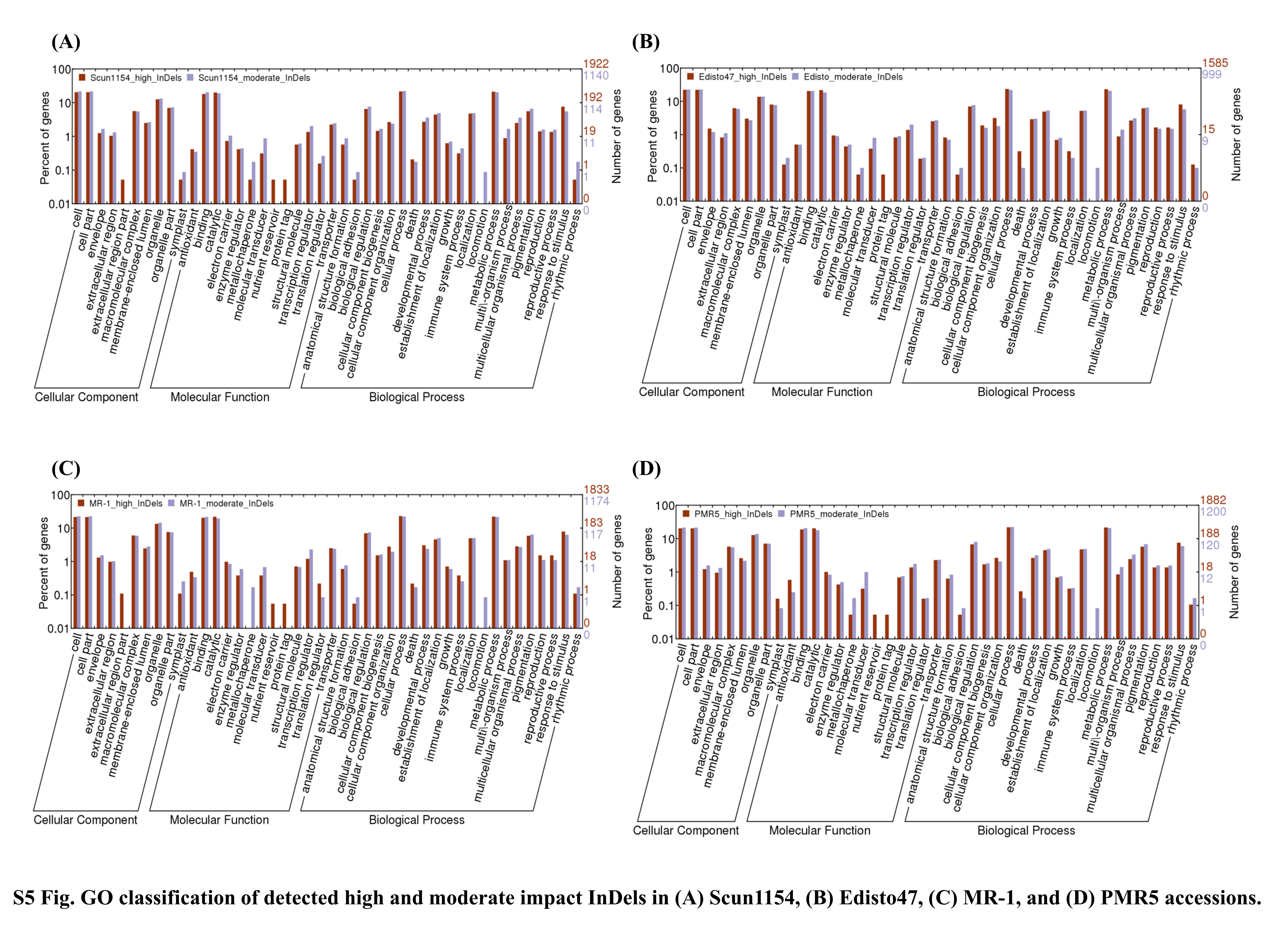

Supplement: S5 Fig — GO classification of detected high- and moderate-effect InDels in (A) SCNU1154, (B) Edisto47, (C) MR-1, and (D) PMR5 accessions. (TIF) [file pone.0157524.s005.tif]

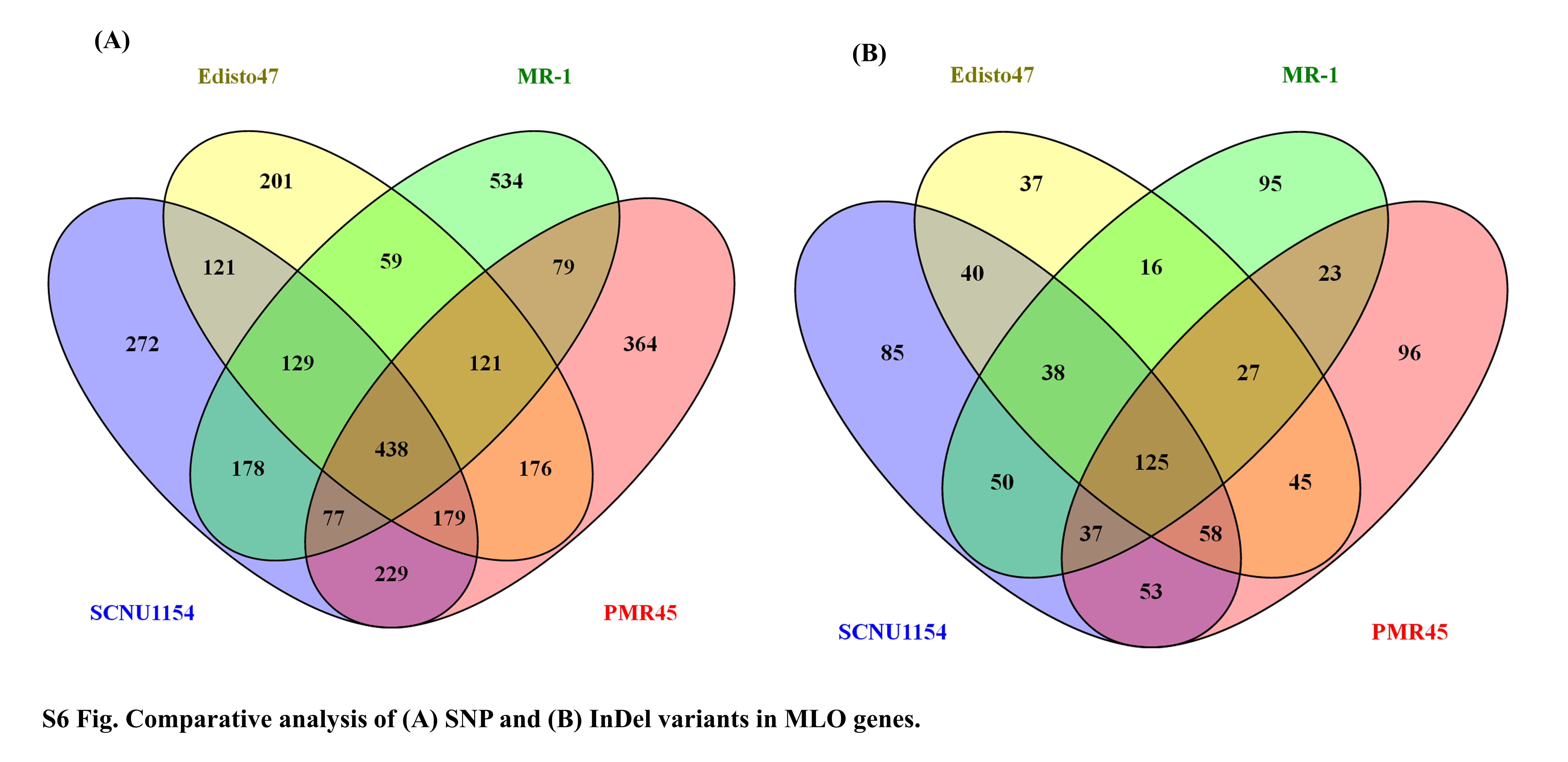

Supplement: S6 Fig — Comparative analysis of (A) SNP and (B) InDel variants in MLO genes. (TIF) [file pone.0157524.s006.tif]
